# Analysis and Correction of the Shrinkage Prediction Model for Manufactured Sand Concrete

**DOI:** 10.3390/ma18163802

**Published:** 2025-08-13

**Authors:** Wei Fan, Yang Wei, Jiyang Yi, Kang Zhao, Binrong Zhu, Guofen Li

**Affiliations:** 1 College of Civil Engineering, Nanjing Forestry University, Nanjing 210037, China; fw@njfu.edu.cn (W.F.); yjyaegean@njfu.edu.cn (J.Y.); zbr2022@njfu.edu.cn (B.Z.); lgf@njfu.edu.cn (G.L.); 2 Jiangsu Provincial College Key Laboratory of Intelligent Bridge Construction and Safe Operation & Maintenance, Nanjing 210037, China

**Keywords:** manufactured sand concrete (MSC), shrinkage, admixture mitigation, model calibration and analysis

## Abstract

With the continuous depletion of natural river sand resources and the escalating ecological degradation caused by excessive sand mining, manufactured sand has emerged as a sustainable and environmentally favorable alternative aggregate, playing an increasingly important role in the advancement of green construction materials. Nevertheless, the shrinkage behavior of manufactured sand concrete (MSC) exhibits significant deviations from that of conventional natural sand concrete due to differences in the material characteristics. Existing shrinkage prediction models—such as ACI 209, CEB-FIP 2010, B3, and GL 2000—fail to adequately incorporate the specific properties and substitution effects of manufactured sand, thereby limiting their predictive accuracy and applicability. To bridge this gap, the present study conducted a systematic evaluation of the four aforementioned classical shrinkage prediction models based on experimental data from MSC specimens incorporating varying replacement rates of manufactured sand. The findings revealed that models such as B3 and CEB-FIP 2010 neglected the influence of critical characteristics of manufactured sand—namely, particle morphology, gradation, and stone powder content—on the cementitious matrix and interfacial transition zone, which led to substantial prediction discrepancies. Accordingly, a nonlinear regression-based correction function was developed, introducing the manufactured sand content as a key influencing variable to recalibrate and enhance the ACI 209 and GL 2000 models for a more accurate application to MSC. The modified models exhibited markedly improved fitting performance and predictive robustness across the full range of manufactured sand replacement ratios (0–100%), thereby offering a more reliable framework for modeling the shrinkage development of MSC.

## 1. Introduction

With the rapid development of the construction industry, manufactured sand (MS), due to its abundant availability, controllable quality, and cost-effectiveness, has gradually replaced natural river sand as a commonly used fine aggregate in concrete production. Numerous studies have shown that manufactured sand concrete (MSC) exhibits excellent mechanical properties, often outperforming natural sand concrete in terms of strength [1,2,3]. However, MSC tends to exhibit more pronounced shrinkage and creep deformations over long-term service, posing potential risks to structural deformation control, cracking resistance, and durability—especially in high-performance or mass concrete applications [4,5,6,7].

In concrete materials, shrinkage refers to the volume reduction caused by cement hydration, moisture loss, pore structure evolution, and internal stress redistribution, and it exhibits significant time dependency. The influencing factors are complex, including the water-to-binder ratio, aggregate type and gradation, curing conditions, ambient humidity, and member dimensions, with strong coupling effects among these variables. Due to its rough surface texture, higher specific surface area, and distinct gradation, manufactured sand alters the interfacial transition zone in concrete compared with natural sand, resulting in faster early drying rates and more complex capillary structures, which exacerbate drying shrinkage strain. As an inherent time-dependent property of concrete, shrinkage significantly impacts long-term structural performance such as cracking, deformation, and durability [8,9,10,11,12,13]. Therefore, shrinkage must be carefully considered in the design and construction of concrete structures, particularly in long-span or high-performance applications [14,15].

Currently, various shrinkage prediction models are widely applied in engineering practice including ACI 209, CEB-FIP 2010, B3, and GL2000 [16,17,18,19]. These models differ in their parameter selections and predictive mechanisms. For instance, the ACI 209 model incorporates factors like the sand ratio, slump, and air content, while the B3 model includes the water-to-cement ratio, cement content, sand ratio, and density. The CEB-FIP 2010 model adopts a more comprehensive theoretical framework and broader experimental database, making it applicable to high-strength concrete and variable humidity environments. However, most of these models have been developed based on data from natural sand concrete and show limited applicability to MSC, failing to accurately capture the unique microstructure, water absorption, and shrinkage behavior of MSC.

As modern concrete continues to evolve in terms of material composition and construction techniques, traditional shrinkage theories no longer fully satisfy the design needs of new-generation concretes like MSC. Currently, no universally accepted shrinkage model exists that provides both high adaptability and prediction accuracy across various concrete types. In response, researchers worldwide have conducted extensive experimental studies, using statistical regression, numerical analysis, and computational optimization to develop new shrinkage prediction models [5,20,21,22]. Some have specifically focused on particular materials or conditions, such as high-strength or MSC, to establish targeted models. Nonetheless, there remains a lack of systematic validation and comprehensive data to assess these models’ applicability, prediction errors, and sensitivity to influencing factors in MSC. Most existing studies are limited to basic comparative analyses, lacking large-scale, broadly representative experimental foundations.

Therefore, this study focused on the shrinkage prediction of MSC. Based on a thorough evaluation of the applicability of existing models, a systematic experimental program on MSC shrinkage behavior was conducted. The study emphasizes assessing the prediction accuracy and error trends of various models when applied to MSC and explores potential directions for model improvement and parameter optimization. The ultimate goal is to establish a more adaptive and practically applicable shrinkage prediction model tailored to MSC, providing theoretical support and practical guidance for its structural applications.

## 2. Comparative Analysis of Typical Concrete Shrinkage Prediction Models

### 2.1. ACI 209 Model

The ACI 209 model, developed by the American Concrete Institute, is one of the earliest empirical models for predicting concrete shrinkage and creep. It uses a time-dependent base function modified by a series of empirical correction factors to account for variables such as curing method, ambient relative humidity, member size, slump, air content, and fine aggregate content. However, the model does not explicitly consider the concrete compressive strength, cement type, or supplementary cementitious materials, which limits its applicability to modern high-performance concretes or concretes with alternative materials like manufactured sand. It is more suited for ordinary Portland cement concrete with conventional materials and curing practices, typically within moderate strength ranges [23,24,25,26]. Due to its simplicity, the model is widely used in engineering practice but often underestimates shrinkage in concrete with low strength or unconventional aggregates.

### 2.2. CEB-FIP 2010 Model

The CEB-FIP 2010 model is a systematic update of the CEB-FIP 90 model, proposed by the International Federation for Structural Concrete (fib), aimed at better aligning with the development of modern concrete materials and practical engineering needs. Based on more comprehensive physical mechanisms and thermodynamic principles, the model separates shrinkage into autogenous shrinkage and drying shrinkage, and introduces additional influencing parameters such as the concrete compressive strength, relative humidity, curing conditions, member size, and loading age to enhance its predictive capability for various types of concrete shrinkage behavior. It is applicable to a wide range of conditions including compressive strengths from 20 to 130 MPa, relative humidity from 40% to 100%, temperatures from 5 °C to 30 °C, and loading ages not less than one day. With more scientific and reasonable parameter settings, the CEB-FIP 2010 model generally provides better prediction accuracy in high-performance concrete than the CEB-FIP 90 and other traditional models [27,28,29]. However, since the model is still primarily based on data from natural sand concrete, its response mechanisms remain insufficient for MSC, especially under early-age drying, interfacial structure changes, and variations in stone powder content, limiting its applicability and accuracy in such cases.

### 2.3. B3 Model

The B3 model, proposed by Bažant and his team, is a comprehensive semi-empirical model developed from an extensive global database of experimental shrinkage and creep results. It incorporates a detailed mechanistic framework that separates basic and drying shrinkage components and introduces corrections for the water–cement ratio, cement content, concrete density, and aggregate volume. The model’s theoretical foundation lies in the microprestress-solidification theory, which better represents the physical processes underlying shrinkage. This makes it one of the most accurate models available for conventional concretes. However, its complexity and data input requirements can be a barrier to practical engineering applications [30,31,32]. Moreover, as its calibration data are mainly derived from natural sand concrete, its predictive accuracy may decrease when applied to concretes with non-traditional aggregates such as manufactured sand, which exhibit different pore structures and moisture transport mechanisms.

### 2.4. GL2000 Model

The GL2000 model, developed by Gardner and Lockman, offers a balanced empirical framework that improves upon the simplicity of ACI 209 while introducing parameters more relevant to modern concrete. It incorporates the effects of concrete strength, member thickness, and curing method, aiming to provide more realistic predictions for both normal and high-strength concretes. Its time function for shrinkage evolution is flexible and can be adapted for varying environmental conditions. Despite this, the model still assumes a relatively standard material composition, and like other models, it lacks specific parameters addressing the microstructural features of MSC. Its utility in predicting shrinkage for MSC is therefore limited unless recalibrated with experimental data reflecting the influence of fine particles, surface texture, and water absorption of the manufactured sand aggregates [33,34].

## 3. Comparative Analysis Between Shrinkage Prediction Models and Experimental Results for MSC

### 3.1. Overview of Experimental Data and Methodology

Although models such as ACI 209, CEB-FIP 2010, B3, and GL2000 have been widely applied in predicting shrinkage in conventional concrete, their theoretical foundations and parameter settings are primarily based on data from natural sand concrete. As a result, their applicability to manufactured sand concrete (MSC)—a widely adopted system—remains limited and inconsistent. The previous section provided a systematic review and comparative analysis of these four models, highlighting potential inaccuracies and missing mechanisms when applied to MSC shrinkage prediction. To enhance the applicability and accuracy of these models in practical MSC applications, the study conducted a comparative analysis between the predicted results of the four models and the actual measured data based on the shrinkage test results obtained from self-performed experiments on MSC. Through parameter adjustment and mechanism enhancement, the models were modified and optimized to more accurately capture the shrinkage behavior of MSC. The following sections detail the development of the experimental database, evaluation of the original models’ suitability, the formulation of correction strategies, and the verification of the revised models.

To compare and modify the existing models, this study conducted actual shrinkage tests on MSC. The experimental conditions were consistent with those used for the model simulations including prismatic specimens with dimensions of 100 × 100 × 400 mm, a uniform curing age of 3 days, and a compressive strength *f_cm_* of 30 Mpa (range 28.7 Mpa to 31.4 Mpa). The environmental conditions for shrinkage tests was as follows: temperature of 20 ± 2 °C (per ASTM C157 [35]) and a relative humidity of 60 ± 10%.

**Figure 1:**
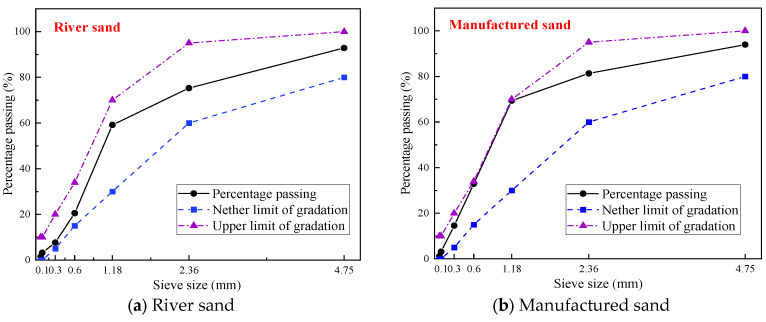
Grading curves of the manufactured sand and river sand.

Ordinary Portland cement (P⋅II 52.5) was used as the binder, and a high-performance, slow-coalescing carboxylic acid-based water-reducing agent was employed as the admixture. Both manufactured sand and river sand were used as fine aggregates. River sand was sourced locally from Nanjing, China, with a fineness modulus between 2.4 and 3.0 and a mud content below 2.0%. Manufactured sand was produced from basalt in Lishui, Nanjing, China. Its particle size distribution is illustrated in Figure 1. Coarse aggregate is made from stones with a maximum particle size less than 25 mm and dense porosity less than 40% respectively. The specific concrete mix is provided in Table 1.

**Figure 2:**
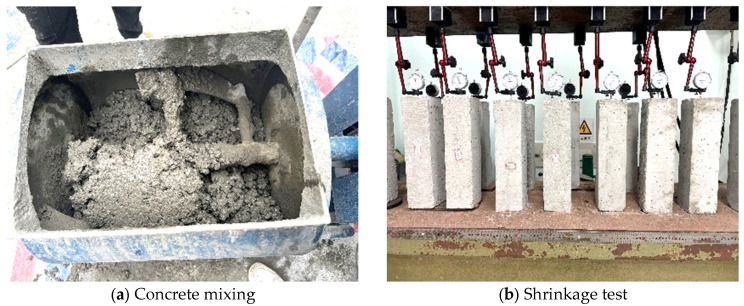
Test process of the concrete specimen.

Table 2 shows the properties of the manufactured sand and river sand used in this study, Table 3 shows the specific mix ratio of the materials used in the concrete casting experiment, and Figure 2 is a photograph of the experiment.

### 3.2. MSC Shrinkage Test Result

**Figure 3:**
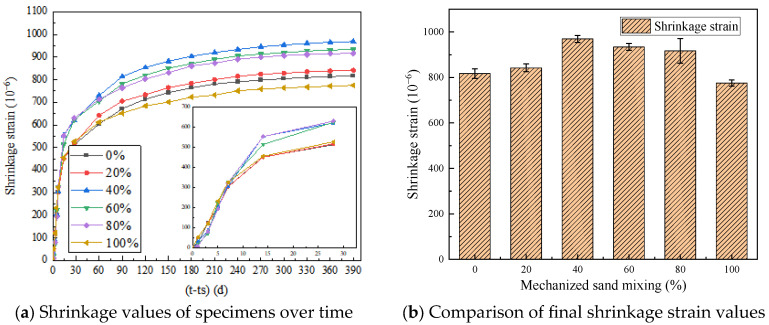
Effect of different replacement rates of manufactured sand on concrete shrinkage.

**Figure 4:**
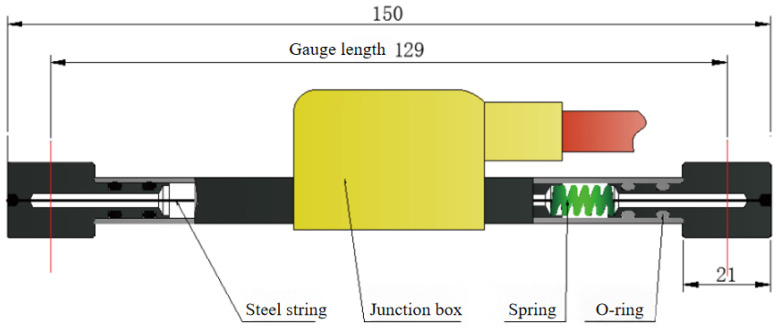
Schematic diagram of vibrating wire strain gauge.

Figure 3 illustrates the time-dependent variation of shrinkage strain for concrete specimens with varying manufactured sand replacement ratios under ambient temperature and humidity conditions. During the monitoring phase, both dial gauges and vibrating wire strain gauges (Figure 4) were used concurrently to capture the strain data.

The strain measurement scheme utilized vibrating wire strain gauges, designed and manufactured by Changsha Jinma Measurement and Control Technology Co., Ltd. (Changsha, China), to measure strain. The specifications of the strain gauges are as follows:

(1) Strain measurement range: ±2500 με; (2) Strain measurement accuracy: 0.5% F.S.; (3) Strain resolution: 0.1 με; (4) Gauge length: 129 mm; (5) Temperature measurement range: −40 °C to +90 °C; (6) Temperature measurement accuracy: ±0.5 °C; (7) Thermal expansion coefficient of steel wire: 12.2 με/°C.

Shrinkage tests on manufactured sand concrete (MSC) with varying replacement ratios showed 270-day maximum shrinkage strains (microstrains, με) as follows: MSC100 (759 με), MSC80 (900 με), MSC60 (914 με), MSC40 (946 με), MSC20 (825 με), and conventional concrete (MSC0, 798 με). MSC40 exhibited the highest shrinkage, while MSC100 had the lowest among the MSC groups. MSC80 showed the highest relative error (10.2%), and MSC0 the lowest (4.4%).

**Figure 5:**
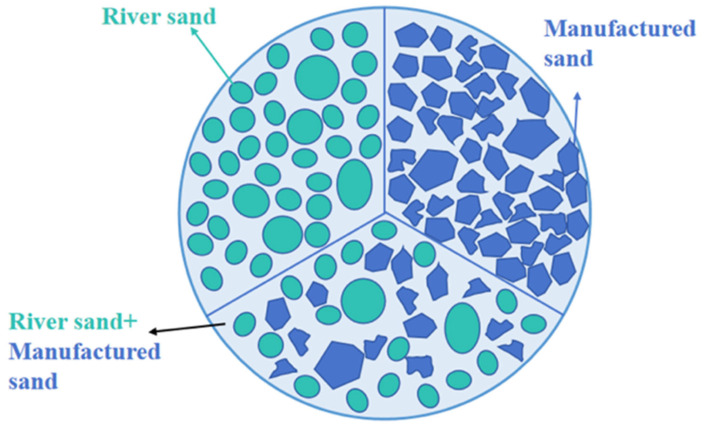
Schematic of different pores formed in cement paste after mixing different types of sand.

Combining manufactured sand with river sand tends to increase the shrinkage strain. This is attributed to the rough surface texture and irregular particle shape of manufactured sand, which accelerate moisture evaporation compared with the smoother texture of river sand. Additionally, mixing two different types of sand increases the likelihood of void formation in the concrete matrix, which promotes shrinkage during hardening. As shown in Figure 1, the gradation curves of the manufactured and river sand presented in the previous section indicate inconsistent gradation patterns and uneven particle size distribution. Therefore, their combination may introduce additional voids in the concrete matrix, further increasing shrinkage during hardening. In contrast, using a single type of fine aggregate helps reduce shrinkage-prone voids in the concrete, thereby mitigating overall shrinkage. Figure 5 below shows the schematic of different pores formed in cement paste after mixing different types of sand.

**Figure 6:**
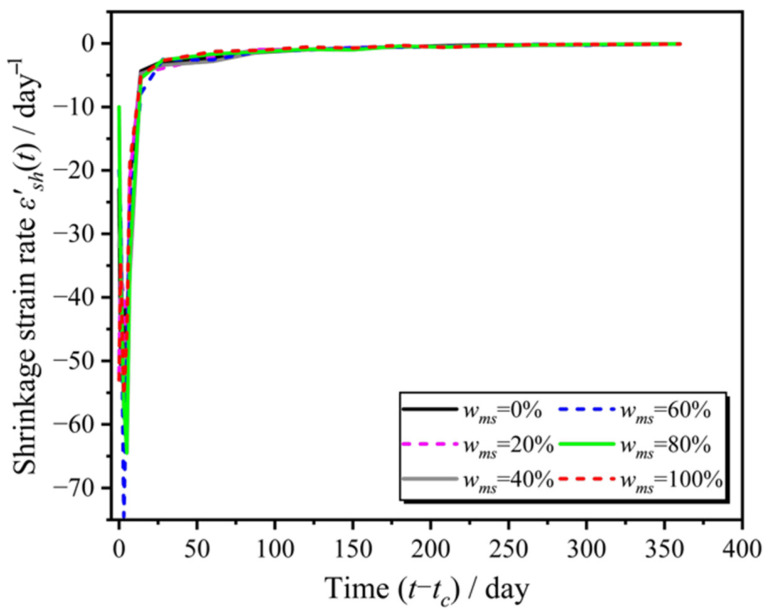
MSC shrinkage strain rate with different replacement rates of manufactured sand.

To address the trend of shrinkage strain, this paper defines a new expression, termed as the shrinkage strain rate, shown as Figure 6, which reflects the intensity of the shrinkage response varying with the independent variable time *t*, given by the following formula:

(1) $${\varepsilon^{\prime }}_{sh}\left(t\right)=\frac{d\varepsilon_{sh}\left(t\right)}{dt}$$

**Figure 7:**
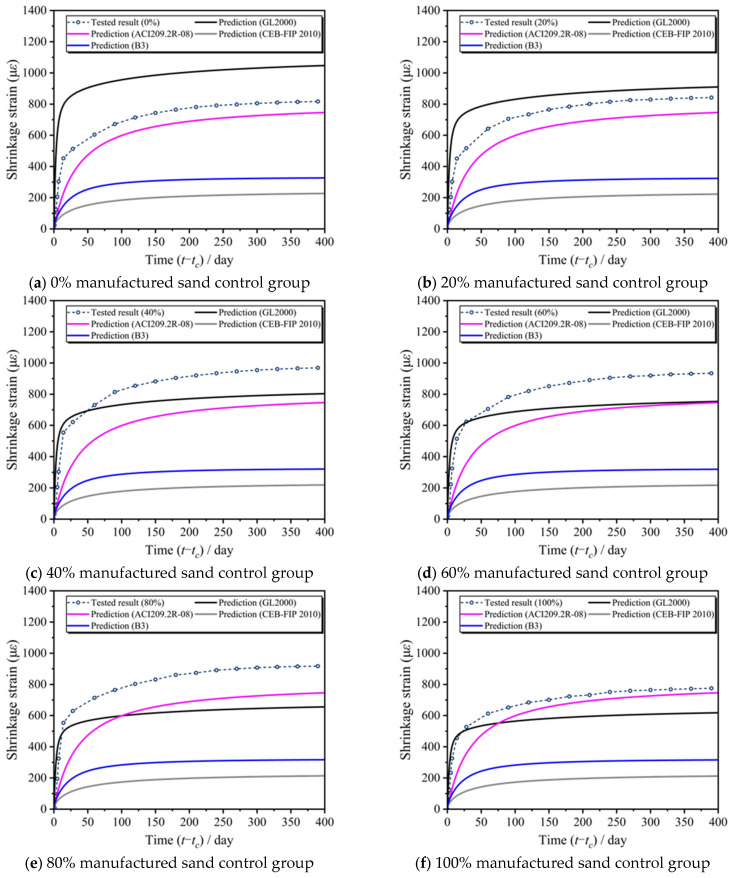
Comparison of the measured values from the MSC shrinkage tests and classical models.

The calculation results of the shrinkage strain rate for different manufactured sand replacement rates are shown in the Figure 7.

### 3.3. Identification of Prediction Deviations in Existing Models

To evaluate the applicability and predictive accuracy of existing concrete shrinkage models for manufactured sand concrete (MSC), this study compared the theoretical predictions from four representative models—ACI 209, CEB-FIP 2010, B3, and GL2000—with the experimentally measured shrinkage strain data under different manufactured sand replacement ratios (0–100%). As shown in Figure 7, the shrinkage tests were conducted under standard ambient temperature and humidity conditions. Due to the limitations of the models, differences in the cement and aggregate used in the experiment, and boundary effects, deviations occurred in the shrinkage prediction of conventional concrete. The results indicate that all four models exhibited varying degrees of deviation from the measured values. Among them, the ACI 209 and GL2000 models showed relatively good agreement with the overall shrinkage trend observed in the experiments, whereas the B3 model, despite accounting for a greater number of parameters, significantly underestimated the total shrinkage throughout the test period. The CEB-FIP 2010 model, in particular, exhibited a notable mismatch in predicting long-term shrinkage, especially during the mid-to-late stages, where the predicted values were substantially lower than those measured in the MSC specimens. Nonetheless, even the relatively better-performing ACI 209 and GL2000 models showed certain errors, highlighting the need for targeted modifications to improve their adaptability to the shrinkage behavior of manufactured sand concrete.

## 4. Analysis and Modification of MSC Shrinkage Models

Based on the comparative analysis between the theoretical models and experimental data, it is evident that the B3 and CEB-FIP 2010 models showed significant deviations from the measured shrinkage values of manufactured sand concrete (MSC). The discrepancies were too large to justify meaningful correction or fitting. In contrast, the ACI 209 and GL2000 models demonstrated a much closer alignment with the overall shrinkage trends and performed better across different manufactured sand replacement levels [39,40]. Therefore, this study selected the ACI 209 and GL2000 shrinkage models for further modification and fitting to improve their predictive accuracy for MSC.

### 4.1. B3 Model

The B3 model calculates concrete shrinkage by dividing it into two components: drying shrinkage and autogenous shrinkage. The main calculation formulas are as follows:

(2) $$\varepsilon_{sh}\left(t,t_{s}\right)=\varepsilon_{sh0}(t)+\varepsilon_{shD}\left(t,t_{s}\right)$$

where *ε_sh_*(*t*,*t_s_*) is the total shrinkage strain from the time *t_s_* when drying begins to the current time *t*; *ε_sh_*_0_(*t*) is the autogenous shrinkage; *ε_shD_*(*t*,*t_s_*) is the drying shrinkage.

The calculation method for autogenous shrinkage *ε_sh_*_0_(*t*) is as follows:

(3) $$\varepsilon_{sh0}(t)=\varepsilon_{sh\infty }\cdot \left(\frac{t}{t+t_{0}}\right)$$

where *ε_sh_*_∞_ is the ultimate autogenous shrinkage strain, with an empirical value of approximately 2.5 × 10^−4^; *t*_0_ is the time constant controlling the development of shrinkage, the recommended value of which is about 1–3 day; *t* is the age (in days).

The method for calculating drying shrinkage *ε_shD_*(*t*,*t_s_*) in concrete according to the B3 model is introduced in the following section.

(4) $$\varepsilon_{shD}\left(t,t_{s}\right)=\varepsilon_{shD\infty }\cdot {\left(\frac{t-t_{s}}{\left(t-t_{s}\right)+\tau_{sh}}\right)}^{n}$$

where *ε_shD_*_∞_ is the ultimate value of drying shrinkage; *τ_sh_* is the time constant, related to ambient humidity and effective thickness; and *t_s_* is the time when drying starts.

The B3 model considers multiple factors such as humidity, age, and material properties in shrinkage prediction, making its theoretical basis relatively comprehensive. However, the model parameters are complex and difficult to obtain accurately, which can lead to significant deviations in the prediction results, in practical applications. Additionally, the B3 model does not describe early-age drying shrinkage in enough detail and cannot well capture the rapid development characteristics of early shrinkage.

### 4.2. CEB-FIP 2010 Model

The CEB-FIP 2010 model calculates concrete shrinkage by dividing it into two parts: drying shrinkage and autogenous shrinkage. The main calculation formulas are as follows:

(5) $$\varepsilon_{cs}(t)=\varepsilon_{cd}\left(t,t_{0}\right)+\varepsilon_{ca}(t)$$

where *ε_cs_*(*t*) is the total shrinkage; *ε_cd_*(*t*,*t*_0_) is the drying shrinkage; *ε_ca_*(*t*) is the autogenous shrinkage; *t*_0_ is the time when drying begins (usually the age at demolding)*; t* is the age after the start of the experiment (in days).

The calculation method for drying shrinkage *ε_cd_*(*t*,*t*_0_) in the CEB-FIP 2010 model is as follows:

(6) $$\varepsilon_{cd}\left(t,t_{0}\right)=\varepsilon_{cd,0}\cdot \beta_{ds}\left(t-t_{0}\right)$$

where *ε_cd_*_,0_ = *β_RH_*·*ε_cd_*_,∞_ is the final value of drying shrinkage and *β_RH_* = 1.55·(1 − *RH*)^3^ is the relative humidity influence factor (*RH* expressed as a decimal).

(7) $$\varepsilon_{cd,\infty }=1000\cdot \left(\frac{220+110\cdot \alpha_{ds1}}{f_{cm}^{0.5}}\right)\cdot 10^{-6}$$

where *f_cm_* is the mean compressive strength of concrete (MPa) and *α_ds_*_1_ is the coefficient related to cement type.

The drying development function *β_ds_*(*t* − *t*_0_) is expressed as:

(8) $$\beta_{ds}\left(t-t_{0}\right)=\frac{\left(t-t_{0}\right)}{\left(t-t_{0}\right)+0.04\cdot h^{2}}$$

where *h* is the effective thickness of the element (mm).

The expression for concrete autogenous shrinkage in CEB-FIP 2010 is:

(9) $$\varepsilon_{ca}(t)=\varepsilon_{ca,\infty }\cdot \left[1-e^{-0.2\cdot t^{0.5}}\right]$$

In the formula, $\varepsilon_{ca,\infty }$ can be expressed as:

(10) $$\varepsilon_{ca,\infty }=2.5\cdot \left(\alpha_{ds2}-0.12\cdot f_{cm}\right)\cdot 10^{-6}$$

where *α_ds_*_2_ is an empirical coefficient influenced by the water–cement ratio, cement type, and other factors.

The CEB-FIP 2010 model offers a simple approach to shrinkage prediction and is suitable for conventional concrete but is based on standard curing conditions and cannot accurately reflect the shrinkage behavior under varying environmental humidity or non-standard curing. Moreover, the model insufficiently accounts for the shrinkage characteristics of concrete containing mineral admixtures or high-performance concrete, resulting in larger prediction errors under these conditions.

Based on the experimental results in this study, the ACI 209 and GL2000 models provide shrinkage predictions that are more consistent with the behavior of manufactured sand concrete (MSC). In the following section, the shrinkage prediction model applicable to manufactured sand concrete will be derived based on the existing GL2000 model and ACI 209 model.

### 4.3. GL2000 Model

In the calculation of total shrinkage, the GL2000 model was adopted, dividing the shrinkage strain calculation into drying shrinkage *ε_sh_*_,*Aut*_ and autogenous shrinkage *ε_sh_*_,*Dry*_.

(11) $$\varepsilon_{sh}\left(t\right)=\varepsilon_{sh,Aut.}\left(t\right)+\varepsilon_{sh,Dry}\left(t\right)$$

The calculation of drying shrinkage is divided into the ultimate value of autogenous shrinkage strain *ε_shu_*_,*Aut.*_ and a time-varying coefficient *β_Aut._*(*t*).

(12) $$\varepsilon_{sh,Aut.}\left(t\right)=\varepsilon_{shu,Aut.}\cdot \beta_{Aut.}\left(t\right)$$

Their calculation formulas are shown as follows:

(13) $$\varepsilon_{shu,Aut.}=600\cdot {\left(\frac{30}{f_{cm28}}\right)}^{2.5}\cdot 10^{-6}$$

where *f_cm_*_28_ is the compressive strength of the concrete cylinder under standard curing at 28 days. The calculation method for the time development coefficient *β_Aut_*(*t*) is shown as follows:

(14) $$\beta_{Aut.}\left(t\right)=1-e^{-0.1\cdot \sqrt{\frac{t-t_{0}}{t_{1}}}}$$

where *β_Aut._*(*t*) is calculated from the initial concrete specimen *t*_0_ and the time correction factor *t*_1_ (this time correction factor adjusts the time scale relative to the initial setting time *t*_0_ and test time *t*. By assuming *t*_1_ = 1, the model eliminates the need for additional calibration of the time effect, making it practical for standard concrete under typical conditions) together with the test time *t.*

The drying shrinkage component *ε_sh_*_,*Dry*_ is calculated by multiplying the ultimate drying shrinkage strain *ε_shu_*_,*Dry*_, the geometric control factor *β_G_*, the time control factor *β_t_*(*t*), and the age correction factor *γ_tc_*.

(15) $$\varepsilon_{sh,Dry}\left(t\right)=\varepsilon_{shu,Dry}\cdot \beta_{G}\cdot \beta_{t}\left(t\right)\cdot \gamma_{t_{c}}$$

The drying shrinkage ultimate strain *ε_shu_*_,*Dry*_ is calculated as shown below:

(16) $$\varepsilon_{shu,Dry}=900\cdot {\left(\frac{30}{f_{cm28}}\right)}^{2.5}\cdot \kappa_{h}\cdot 10^{-6}$$

where *κ_h_* represents the control factor dominated by humidity (*h*, which is known as *RH*%). The calculations are shown below:

(17) $$\kappa_{h}=\left\{\begin{array}{ccc} 1.0 & , & if\;h\leq 0.4\\ 1-1.18h^{4} & , & if\;0.4<h<0.85\\ 0.35 & , & if\;h\geq 0.85 \end{array}\right.$$

The formula for calculating the geometric control factor is shown below:

(18) $$\beta_{G}={\left(1+1.13\cdot e^{-0.0213\frac{V}{S}}\right)}^{-1}$$

where *β_G_* is defined by *V*, which represents the volume of components, and *S* represents the surface area of components. The time correction factor is a coefficient controlled by the combination of time *t*, curing age *t_c_*, specimen volume *V*, and surface area *S*.

(19) $$\beta_{t}\left(t\right)=\frac{t-t_{c}}{\sqrt{{\left(t-t_{c}\right)}^{2}+0.035{\left(V/S\right)}^{2}}}$$

The curing age correction factor *γ_tc_* is a coefficient of variation dominated by the curing age *t_c_*.

(20) $$\gamma_{t_{c}}=1.0+\frac{10}{t_{c}}\leq 5$$

To address the shrinkage characteristics of MSC, we added the manufactured sand replacement *w_ms_* as a variation coefficient based on the GL2000 model formula.

(21) $$\varepsilon_{sh,w_{ms}}{\left(t\right)}_{\mathrm{GL}}=\varepsilon_{shu,Aut.}\cdot \beta_{Aut.}\left(t\right)+\varepsilon_{shu,Dry}\cdot \beta_{G}\cdot \beta_{t}\left(t\right)\cdot \gamma_{t_{c}}$$

(22) $$\eta_{ms}\left(t,w_{ms}\right)=\frac{\varepsilon_{sh,w_{ms}}\left(t,w_{ms}\right)}{\varepsilon_{sh,w_{ms}}{\left(t\right)}_{\mathrm{GL}}}$$

where *w_ms_* represents the percentage of river sand replaced by manufactured sand used in the MSC, dividing the volume of manufactured sand by the volume of all sand used.

Based on the replacement ratio of manufactured sand, the model correction factor *η_ms_* can be calculated by sequentially substituting the experimental parameter data obtained above, and this factor is related to the replacement ratio of manufactured sand and the number of experimental days *t*.

(23) $$\eta_{ms}\left(t,w_{ms}\right)=\frac{0.0133}{0.0133w_{ms}^{2}-0.0208w_{ms}+t^{-1.974}+0.0182}$$

By taking all the above values into Equation (24), the predicted concrete shrinkage of MSC can be obtained for any number of test days and any replacement rate. This process is based on the revised GL2000 shrinkage model, where the replacement ratio of manufactured sand and experimental time are used as independent variables for parameter substitution and fitting with the experimental data, thereby ensuring that the model can accurately describe and predict the shrinkage characteristics of concrete under different replacement ratios and time conditions.

(24) $$\varepsilon_{sh,w_{ms}}\left(t,w_{ms}\right)=\eta_{ms}\left(t,w_{ms}\right)\cdot \varepsilon_{sh,w_{ms}}{\left(t\right)}_{\mathrm{GL}}$$

**Figure 8:**
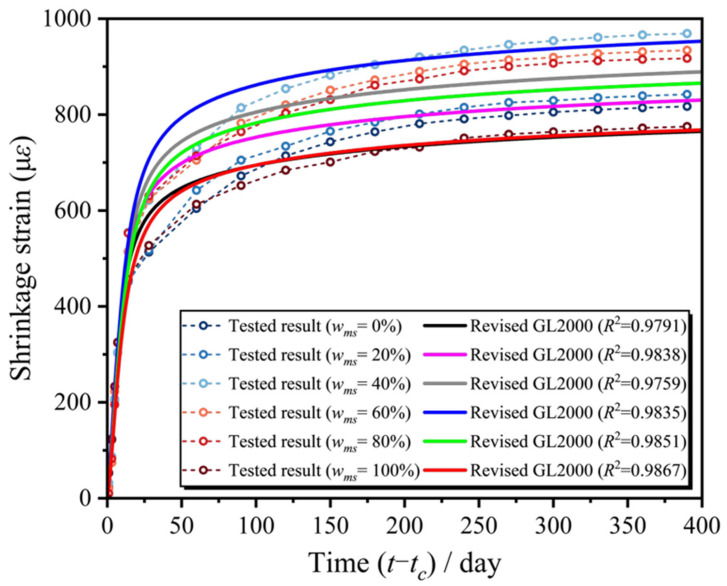
Comparison of the corrected GL2000 model and MSC test values.

Figure 8 was obtained by comparing the calculated values of the revised GL2000 prediction model with the measured values of the test.

### 4.4. ACI 209 Model

Similarly, the ACI 209 model calculates the shrinkage strain by dividing it into two parts: autogenous shrinkage and drying shrinkage:

(25) $$\varepsilon_{sh}\left(t\right)=\varepsilon_{sh,Aut.}\left(t\right)+\varepsilon_{sh,Dry}\left(t\right)$$

where *ε_sh,Aut._*(*t*) is calculated by the formula below:

(26) $$\varepsilon_{sh,Aut.}\left(t\right)=\varepsilon_{shu,Aut.}\cdot \frac{t}{g+t}$$

In the formula, *g* is the time constant, taken as 35 days. This parameter governs the rate at which drying shrinkage develops and is typically derived from the experimental data for concrete with a curing age of 3 days and standard volume-to-surface area ratios (*V*/*S*). The choice of *g* = 35 days is based on empirical calibrations that assume typical concrete properties and environmental conditions, ensuring that the model’s predictions align with the observed shrinkage behavior in practice. The calculation of ultimate autogenous shrinkage is consistent with the GL2000 model.

(27) $$\varepsilon_{shu,Aut.}=600\cdot {\left(\frac{30}{f_{cm28}}\right)}^{2.5}\cdot 10^{-6}$$

where *ε_sh,Dry_*(*t*) is calculated by the formula below:

(28) $$\varepsilon_{sh,Dry}\left(t\right)=\varepsilon_{shu,Dry}\cdot \frac{t-t_{c}}{f+\left(t-t_{c}\right)}\cdot \gamma_{sh}$$

In the formula, *f* is the time constant, which depends on the size of the component and the type of curing. For steam curing, it is taken as 55, while for wet curing, it is given by the following formula:

(29) $$f=26.0\cdot e^{0.36\cdot \frac{V/S}{25.4}}$$

where the definitions of *V* and *S* are the same as in the GL2000 model.

(30) $$\gamma_{sh}=\gamma_{t_{c}}\cdot \gamma_{RH}\cdot \gamma_{V/S}\cdot \gamma_{s}\cdot \gamma_{\varphi }\cdot \gamma_{c}\cdot \gamma_{a}$$

The total correction factor *γ_sh_* comprehensively considers various influencing factors including the curing age factor, relative humidity factor, volume-to-surface area ratio factor, slump factor, fine aggregate proportion factor, cement content factor, and air content factor.

(31) $$\gamma_{t_{c}}=1.10-0.017\cdot t_{c}$$

where *t_c_* is the curing duration (days).

(32) $$\gamma_{RH}=\left\{\begin{array}{ccc} 1.40-0.010\cdot RH & , & if\;40\leq RH\leq 80\\ 3.00-0.030\cdot RH & , & if\;80<RH\leq 100 \end{array}\right.$$

*RH* is the ambient relative humidity (%). The higher the humidity, the smaller the shrinkage strain.

(33) $$\gamma_{V/S}=1.2\cdot e^{-0.00472\cdot \frac{V/S}{25.4}}$$

*V*/*S* is the volume-to-surface area ratio of the component (inches).

(34) $$\gamma_{s}=0.89+0.041\cdot s$$

*s* is the slump of the concrete, measured in inches (in.).

(35) $$\gamma_{\varphi }=\left\{\begin{array}{ccc} 0.30+0.014\cdot \varphi & , & \varphi \leq 50\\ 1.0 & , & \varphi >50 \end{array}\right.$$

*φ* is the weight percentage of fine aggregate in the total aggregate (%).

(36) $$\gamma_{c}=0.75+0.00061\cdot c$$

Cement component in the research was 395 kg/m^3^, the cement content factor *γ_c_* was calculated as 0.9910, the fine aggregate proportion factor *γ_φ_* was calculated as 0.8757, and the slump factor *γ_s_* and air content factor *γ_a_* were taken as 1. The total calculation formula of the ACI 209 model is as follows:

(37) $$\varepsilon_{sh,w_{ms}}{\left(t\right)}_{\mathrm{ACI}}=\varepsilon_{shu,Aut.}\cdot \frac{t}{g+t}+\varepsilon_{shu,Dry}\cdot \frac{t-t_{c}}{f+\left(t-t_{c}\right)}\cdot \gamma_{sh}$$

To address the shrinkage characteristics of MSC, we added the manufactured sand replacement *w_ms_* as a variation coefficient based on the ACI 209 model formula.

(38) $${\eta^{\prime }}_{ms}\left(t,w_{ms}\right)=\frac{\varepsilon_{sh,w_{ms}}\left(t,w_{ms}\right)}{\varepsilon_{sh,w_{ms}}{\left(t\right)}_{\mathrm{ACI}}}$$

By using the replacement ratio of manufactured sand and sequentially substituting the obtained experimental parameter data, the model correction factor *η*′*_ms_* can be calculated, which is associated with the replacement ratio of manufactured sand and the number of experimental days *t*.

(39) $${\eta^{\prime }}_{ms}\left(t,w_{ms}\right)=\frac{38.62+88.30\cdot t^{-0.4045}}{44.76+28.76w_{ms}^{2}-29.49w_{ms}}$$

By substituting all the aforementioned values into Equation (40), the anticipated shrinkage of MSC can be determined for any duration of testing days and any replacement ratio. This approach relies on the updated ACI shrinkage model, which utilizes the manufactured sand replacement ratio and testing duration as independent variables for parameter substitution and alignment with the experimental data, thus ensuring that the model accurately captures and forecasts the concrete’s shrinkage behavior across varying replacement ratios and time periods.

(40) $$\varepsilon_{sh,w_{ms}}\left(t,w_{ms}\right)={\eta^{\prime }}_{ms}\left(t,w_{ms}\right)\cdot \varepsilon_{sh,w_{ms}}{\left(t\right)}_{\mathrm{ACI}}$$

**Figure 9:**
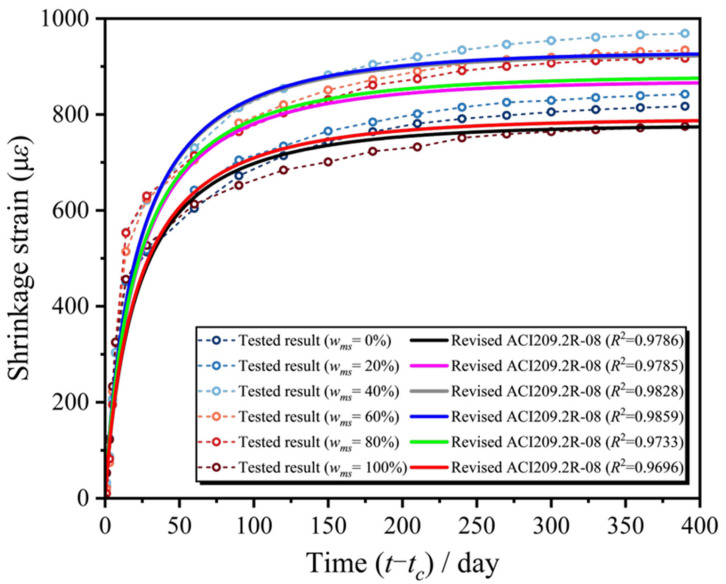
Comparison of the corrected ACI 209 model and MSC test values.

Figure 9 was obtained by comparing the calculated values of the revised ACI209 prediction model with the measured values of the test. Figure 9 presents a comparison between the predicted values derived from the revised ACI 209 shrinkage and creep prediction model and the actual measured values obtained from the experimental tests. The results demonstrate that the revised ACI 209 model yields predictions that closely align with the observed engineering data. This high degree of agreement indicates the model’s reliability and accuracy in capturing the time-dependent behavior of concrete, such as shrinkage, under typical conditions. The close correspondence between the calculated and measured values underscores the effectiveness of the model’s revisions, making it a robust tool for practical engineering applications where the precise prediction of concrete deformation is essential.

Table 4 compares the accuracy of different models in predicting the shrinkage behavior of MSC (manufactured sand concrete). The CEB-FIP 2010 and B3 models had negative R^2^ values, indicating extremely poor prediction performance and complete unsuitability for MSC shrinkage prediction. The GL2000 model exhibited R^2^ values from 0.0667 to 0.7999, with peak accuracy (R^2^ = 0.7999) at the 40% replacement ratio. The ACI 209.2R-08 model maintained high precision (R^2^ ≥ 0.80) at low replacement ratios (≤30%), though its R^2^ range was 0.6329–0.8669. Both models showed significantly reduced accuracy at high replacement ratios (≥60%) (GL2000: R^2^ ≤ 0.35; ACI 209: R^2^ ≤ 0.65). After incorporating the manufactured sand replacement rate (*w_ms_*) as a correction factor, the R^2^ values of the GL2000 and ACI models significantly improved to 0.9696–0.9859, with accuracy improvements of 22.0–1367.9% and 12.0–55.3%, respectively, demonstrating strong adaptability to varying replacement ratios. The revised GL2000 model slightly outperformed the revised ACI model with higher R^2^ values, making it the best choice for MSC shrinkage prediction to ensure high accuracy and stability.

## 5. Conclusions

This study investigated the variation in shrinkage behavior of concrete with different replacement ratios of manufactured sand for river sand. It systematically analyzed and compared the advantages and limitations of existing concrete shrinkage models and their applicability to manufactured sand concrete (MSC). Based on the experimental results, the GL2000 model and ACI 209 model were modified and improved to develop a shrinkage prediction model suitable for MSC.

1. By comparing the shrinkage test results of MSC with classical shrinkage models, the gap between the predicted values of existing models and the measured shrinkage of MSC was identified. Among them, the B3 and CEB-FIP 2010 models significantly underestimated the shrinkage of MSC, while the ACI 209 and GL2000 models provided overall predictions that were closer to the measured values.
2. Based on the experimental data, the GL2000 and ACI 209 models were modified to improve their shrinkage prediction for MSC. The revised models introduced the manufactured sand content as a new variable and showed overall shrinkage curves that more closely matched the actual shrinkage strain behavior of MSC.
3. The research showed that the CEB-FIP 2010 and B3 models performed poorly in predicting the MSC shrinkage (R^2^ negative), while the original GL2000 and ACI 209.2R-08 models had a limited accuracy (R^2^ from 0.0667 to 0.8669). After introducing the manufactured sand dosage correction factor, the R^2^ of GL2000 and ACI models improved to 0.9696–0.9859, with the revised GL2000 model performing best and is recommended for MSC shrinkage prediction.

These findings provide substantial theoretical support for exploring MSC shrinkage behavior, including a comparative analysis of classical shrinkage models to evaluate their advantages, limitations, and applicability, and propose improvements based on existing models. However, a limitation of this study is that all experimental data were based on a single aggregate (basalt), and thus the conclusions are specific to basalt-based MSC. Other aggregate types (e.g., granite, limestone) may influence the shrinkage behavior differently, warranting further research to validate the models’ generalizability. Additionally, MSC shrinkage is significantly influenced by other variables, such as aggregate particle size distribution and environmental conditions, which require further investigation.

## Figures and Tables

**Table 1:** Overview and comparison of classic concrete shrinkage prediction models.

Feature	ACI 209	CEB-FIP 2010	B3	GL2000
Theoretical basis	Empirical	Semi-empirical	Semi-mechanistic	Empirical
Strength inclusion	×	√	√	√
Shrinkage components	Total only	Drying + Autogenous	Basic + Drying	Total only
Key parameters	Sand ratio, slump, RH, size	Strength, cement type, RH, curing	w/c ratio, cement, density, RH, size	Strength, RH, curing, size
Complexity	Low	Medium	High	Medium

Note: “RH” stands for relative humidity, and “w/c” stands for the water–cement ratio.

**Table 2:** Physical properties of fine aggregates.

Parameter	River Sand	MSC	Standard
Apparent density	2.63 ± 0.02 g/cm^3^	2.71 ± 0.03 g/cm^3^	GB/T 14685-2022 [36]
Water absorption	1.0 ± 0.1%	2.3 ± 0.2%	GB/T 17431.2 [37]
Fineness modulus	2.6	3.1	ASTM C136 [38]

**Table 3:** Mix proportion (kg/m^3^).

Strength Grade	Cement	Sand	Coarse Aggregate	Water	Water Reducing Agent
C30	395	810	1160	188	0.5

**Table 4:** Comparison of the accuracy of shrinkage predictions from different models with the MSC measured values.

Manufactured Sand Replacement Ratio	Coefficient of Determination (R^2^)							
	CEB-FIP 2010	B3	GL2000	Revised GL2000	Accuracy Improvement for GL2000	ACI 209	Revised ACI 209	Accuracy Improvement for ACI 209
*w_ms_* = 0%	−1.6643	−0.7602	0.0667	0.9791	1367.9%	0.8669	0.9786	12.9%
*w_ms_* = 20%	−1.7677	−0.8610	0.6878	0.9838	43.0%	0.8372	0.9785	16.9%
*w_ms_* = 40%	−1.8687	−1.0748	0.7999	0.9759	22.0%	0.6329	0.9828	55.3%
*w_ms_* = 60%	−1.8572	−1.0338	0.7816	0.9835	25.8%	0.6854	0.9859	43.8%
*w_ms_* = 80%	−1.8770	−1.0362	0.6210	0.9851	58.6%	0.6954	0.9733	40.0%
*w_ms_* = 100%	−1.9597	−0.9068	0.7865	0.9867	25.5%	0.8658	0.9696	12.0%

Note: R^2^ can take on a negative value when the model provides a worse fit than a horizontal line representing the mean of the data (i.e., when the residual sum of squares (RSS) is greater than the total sum of squares (TSS)).

## Data Availability

The original contributions presented in this study are included in the article. Further inquiries can be directed to the corresponding authors.
